# Nosocomial meningitis caused by *Staphylococcus haemolyticus* in a child with neutropenia in the absence of intracranial devices: a case report

**DOI:** 10.1186/s12879-023-08059-5

**Published:** 2023-02-14

**Authors:** Shinsuke Mizuno, Tomoko Fujikawa, Suguru Uemura, Daiichiro Hasegawa, Yoshiyuki Kosaka, Masashi Kasai

**Affiliations:** 1 Division of Infectious Disease, Department of Pediatrics, Hyogo Prefectural Kobe Children’s Hospital, Kobe, Hyogo Japan; 2 Division of Hematology and Oncology, Department of Pediatrics, Hyogo Prefectural Kobe Children’s Hospital, Kobe, Hyogo Japan; 3 Department of Pediatrics Infectious Disease, Hyogo Prefectural Kobe Children’s Hospital, 1-6-7 Minamimachi, Minatojima, Chuoku, Kobe, Hyogo 6500047 Japan

**Keywords:** Coagulase-negative staphylococcus, Meningitis, Neutropenia, *Staphylococcus haemolyticus*

## Abstract

**Background:**

Coagulase-negative staphylococci can cause hospital-acquired infections, especially in immunocompromised hosts. Bacterial meningitis is a potentially fatal infection of the central nervous system, causing high mortality and morbidity. In general, the causative agents of meningitis, coagulase-negative staphylococci, are associated with direct implantation of a foreign body and the presence of a cerebrospinal fluid (CSF) shunt. Here, we describe a case of nosocomial meningitis caused by *Staphylococcus haemolyticus* in a child with neutropenia who had no intracranial foreign devices.

**Case presentation:**

A 15-year-old boy with relapsed acute myeloid leukemia undergoing chemotherapy through a central venous catheter developed fever on Day 13 post-initiation of chemotherapy. There was no history of implantation of neurosurgical devices. Two blood cultures obtained on Day 14 were positive for *Staphylococcus haemolyticus*. Clinical improvement was noted, and treatment with vancomycin and removal of the central venous catheter resulted in negative repeat blood cultures on Day 18. However, the patient developed a tendency for somnolence and improper speech, along with persistent fever on Day 26. A lumber puncture was performed on Day 27, resulting in positive culture of *Staphylococcus haemolyticus*. He was diagnosed with meningitis and the dosage of vancomycin was increased. A repeat CSF culture was positive for *Staphylococcus haemolyticus* on Day 40, so oral rifampicin was added. CSF findings on Day 46 revealed a low concentration of vancomycin, and treatment was switched from vancomycin plus rifampicin to linezolid. After Day 46, four subsequent cerebrospinal fluid tests of the CSF showed no growth of *Staphylococcus haemolyticus*. The patient’s symptoms were improved on Day 52. Brain and spinal magnetic resonance images was taken and it showed no abnormalities. Linezolid was continued until Day 72. The patient was discharged without any complications on Day 72.

**Conclusions:**

To the best of our knowledge, this is the first reported case of *Staphylococcus haemolyticus* meningitis in a patient without a neurosurgical device. Typical symptoms or signs may be absent in a patient with meningitis who also has neutropenia. Repeated tests of the CSF, and prolonged duration of antibiotics should be considered if atypical pathogens are detected in immunocompromised hosts.

## Background

Cases of meningitis with neutropenia rarely show reliable signs and symptoms, leading to delayed diagnosis and high mortality [1–3]. *Staphylococcus haemolyticus* (*S. haemolyticus*) is a major cause of nosocomial infections; indeed, it is the most common cause of infection in patients with intravenous catheters and indwelling devices [4]. Colonization of the skin of patients and health care workers is the most common source of *S. haemolyticus* infections. Usually, *S. haemolyticus* meningitis is associated with trauma or use of neurosurgical devices; *S. haemolyticus* meningitis in the absence of these devices has never been reported [3]. Here, we report a pediatric case of *S. haemolyticus* meningitis with neutropenia, unrelated to the use of a neurosurgical device. The patient was diagnosed by repeat lumber punctures and treated successfully.

## Case presentation

In April, 2021, a 15-year-old boy was diagnosed with acute myeloid leukemia with RUNX1-RUNX1T1. Cytogenetics revealed t(8;21) (q22;q22) in addition to trisomy 4. He started chemotherapy following the JPLSG AML-12 protocol, which includes high-dose cytarabine as initial induction therapy with combination therapy of the continuous administration of cytarabine. He entered complete remission after induction therapy. His treatment finished in October of that year; however, the leukemia relapsed in January, 2022. He underwent chemotherapy with the relapse protocol, which comprises fludarabine, cytarabine, granulocyte colony-stimulating factor, and idarubicin. In January, 2022, a Hickman catheter was placed to facilitate intravenous chemotherapy. A second course of chemotherapy was started in May, 2022, which resulted in prolonged pancytopenia. His medical history revealed a diagnosis of an epidural tumor, treated by resection and laminectomy in April 2021, followed by multiple cycles of chemotherapy. Postoperatively, no neurosurgical devices were left behind.

On Day 6 post-initiation of chemotherapy, he developed fever, which was treated empirically with intravenous cefepime. At the time, he was alert. His temperature was 40 °C, his pulse was 120 beats per minute, his blood pressure was 97/46 mmHg, and his respiratory rate was 22 breaths per minute. Physical examination was unremarkable, including nuchal rigidity. The patient’s laboratory results were as follows: absolute neutrophil count, < 0.1 × 10^9^/L; platelet count, 36 × 10^9^/L; and C-reactive protein, 1.65 mg/dL (elevated). The fever improved after treatment. On Day 13, the patient again developed fever. Cefepime was switched empirically to meropenem. Two sets of blood cultures were obtained, and both were positive for *S. haemolyticus* (Table 1). After 11 and 14 h of incubation, the blood culture bottle from the central venous catheter and peripheral vein, respectively, were reported positive for *S. heamolyticus*; based on this, we made a diagnosis of catheter-related bloodstream infection (differential time to positivity > 2 h) [5]. Therefore, vancomycin was added and the central venous catheter was removed. Repeat blood culture on Day 18 was negative, despite prolonged fever and neutropenia. On Day 26, the patient developed a tendency to somnolence and impaired speech; the fever persisted, but there was no headache or nuchal rigidity. Magnetic resonance imaging (MRI) of the brain revealed no abnormalities. A lumbar puncture performed on Day 27 revealed clear cerebrospinal fluid (CSF), with 2 cells/μL. CSF glucose levels were slightly low (2.1 mmol/L) and protein levels were normal (3.9 g/L). Gram staining of the CSF did not detect any microorganisms. However, *S. haemolyticus* was isolated from the CSF; these isolates showed the same susceptibility as the isolates from blood cultures (Table 1). On the same day, a complete blood count revealed an absolute neutrophil count of 0.09 × 10^9^/L. At that time, there was no strong suspicion that this organism was the true pathogen. A second lumbar puncture on Day 33 revealed that the total cell count in the CSF was 260/μL, the glucose level was 2.1 mmol/L, and the protein level was 4.4 g/L, indicating a diagnosis of meningitis. No organisms were isolated from blood and CSF cultures. On the same day, a complete blood count revealed an increased absolute neutrophil count of 1.48 × 10^9^/L. We continued to administer vancomycin. A third lumbar puncture performed on Day 40 revealed an improvement in CSF parameters (cell count, 65 cells/μL; glucose, 2.2 mmol/L; and protein, 4.1 g/L), but a CSF culture was positive for *S. haemolyticus*. The antimicrobial susceptibility profile was the same as that for strains detected in the blood and CSF. The diagnosis of meningitis was confirmed, and so oral rifampicin (300 mg every 12 h) was added to the regimen. The vancomycin concentrations in the blood and CSF were measured during treatment. Serum trough concentrations of vancomycin were within the therapeutic range of 13.0 to 22.4 mg/L, with a peak serum concentration of 22.4 mg/L on Day 35. However, the CSF trough level was < 2.5 mg/L on Day 46, so we switched from vancomycin plus rifampicin to intravenous linezolid (600 mg every 12 h). After initiation of linezolid, several lumber puncture tests were performed, which showed no growth after the third lumber puncture. Contrast-enhanced MRI of the brain and spinal cord showed no abnormalities suggestive of a local disturbance of the blood-CSF-barrier, abscess, or ventriculitis during treatment. Antibiotic treatment was continued until Day 72, and the patient was discharged without complications on Day 72.

**Table 1:** Antimicrobial susceptibility profile of the *Staphylococcus haemolyticus* isolated from blood and CSF

Antimicrobial	MIC (mcg/mL)	Interpretation
Ampicillin	> 8	R
Ampicillin/sulbactam	> 8	R
Cefazolin	> 16	R
Cefmetazole	> 16	R
Imipenem	> 8	R
Gentamicin	> 8	R
Erythromycin	> 4	R
Levofloxacin	> 4	R
Sulfamethoxazole/Trimethoprim	> 2	R
Vancomycin	2	S
Teicoplanin	4	S
Linezolid	< 1	S
Daptomycin	< 0.25	S
Clindamycin	< 0.5	S
Minocycline	< 2	S
Rifampicin	< 0.5	S

S: Susceptible; I: Intermediate; R: Resistant

## Discussion and conclusions

Here, we describe a neutropenic patient with *S. haemolyticus* meningitis, unrelated to the use of a neurosurgical device. Although diagnosis of meningitis in neutropenic patients is difficult because they usually present with only a few reliable signs and symptoms, this patient was diagnosed by repeat lumber punctures. In the absence of neurosurgical indwelling devices, CoNS isolated from CSF is considered to be a contaminant; however, the present study shows that CoNS can be a causative agent in patients with neutropenia.

*Streptococcus pneumoniae*, *Staphylococcus aureus*, *Pseudomonas aeruginosa*, and *Escherichia coli* are common causative agents of bacterial meningitis in neutropenic patients [1, 2]. However, to date, there is no documented case of neutropenic meningitis caused by *S. haemolyticus*. A review of 43 cases of neutropenic meningitis revealed that the most consistent clinical manifestations are fever and altered mental status; headache is rare [2], and nuchal rigidity is also uncommon. Compared with non-neutropenic patients with meningitis, those with neutropenia do not present with as many symptoms. The CSF cell counts in neutropenic patients are considerably lower than those in non-neutropenic patients [1, 2]. In our patient, we noted a tendency to somnolence, speech impediment, and persistent fever, but no headache or nuchal rigidity. The only CSF abnormality at the time of the initial lumbar puncture was a slight decrease in glucose level. Subsequent lumbar punctures showed an elevated cell counts in the CSF, with a recovery in the peripheral blood neutrophil counts.

A previous study on CoNS meningitis reported a 30 day mortality rate of 14% after treatment with appropriate antibiotics and removal of the neurosurgical devices [3]. Our patient was treated successfully by prolonged administration of intravenous vancomycin, oral rifampin, intravenous linezolid, and removal of the central venous catheter.

Meningitis caused by *S. haemolyticus* infection is associated almost exclusively with neurosurgical procedures, neurosurgical devices, and head trauma. In a previous review of 561 cases of meningitis caused by CoNS, almost all were post-neurosurgical with insertion of neurosurgical devices [3]. Forty cases of CoNS meningitis were reviewed. The median interval from the last neurosurgical procedure to the diagnosis of CoNS meningitis was 10.5 days [6]. Although our patient had a history of spinal surgery, 12 months had passed since his last neurosurgical procedure, and no neurosurgical device was implanted. Therefore, it is unlikely that meningitis developed from a primary infection at the surgical site. Table 2 lists cases of CoNS meningitis unrelated to the use of neurosurgical devices [3, 7]. All of these patients had an underlying condition associated with their immunity, and all of them had an indwelling foreign body other than an intracranial device that was left in place.

**Table 2:** Characteristics of patients with coagulase-negative *staphylococcus* meningitis in the absence of intracranial devices

No	Age, sex	Underlying conditions	CoNS species	Repeated positive CSF culture	Treatment/Outcome	References
1	20 years, F	CML, BMT, neutropenia	*S. epidermidis*	Yes	Vancomycin/death	Guiot et al. (1994)
2	18 days, F	ELBW, IH	*S. epidermidis*	Yes	Vancomycin/recovery	Drinkovic et al. (2002)
3	20 days, F	ELBW, IH	*S. capitis*, *S. warneri*	Yes	Vancomycin, rifampicin/recovery	Drinkovic et al. (2002)
4	65 years, F	DM, LC	*S. capitis*	No	Vancomycin/death	Oud (2011)
5	11 months, M	Decreased T-cell, count, hypo- gammaglobulinemia	*S. intermedius*	No	Cefotaxime/recovery	Durdik et al. (2010)
6	47 years, M	AML, oligodendrogliaoma	S. epidermidis	No	Vancomycin, rifampicin/recovery	Noguchi et al. (2018)
7	15 years, M	AML, neutropenia	*S. haemolyticus*	Yes	Vancomycin, rifampicin, linezolid/recovery	Our patient

CoNS: Coagulase-negative Staphylococcus; CSF: Cerebrospinal fluid; F: Female; M: Male; CML: Chronic myeloid leukemia; BMT: Bone marrow transplant; ELBW: Extreme low birth weight; AML: Acute myeloid leukemia

In the absence of an indwelling neurosurgical device, CoNS isolated from CSF is generally considered to be a contaminant; Durand et al. suggested that CoNS should be classed as a causative agent only if it is detected repeatedly [8]. This diagnostic criterion was adopted by other investigators [9]. In our patient, *S. haemolyticus* was detected in the first and third CSF tests, although the organism was not detected by Gram staining of the CSF. This might have been because vancomycin was administered prior to lumbar punctures and the inoculum of *S. haemolyticus* was low.

In conclusion, CoNS can cause meningitis in patients with neutropenia in the absence of a neurosurgical device. There are few reliable signs and symptoms in patients with neutropenia. Repeat lumber puncture and prolonged treatment with antibiotics should be considered if atypical pathogens are detected in a patient with neutropenia.

## Data Availability

The datasets used and/or analyzed during the current study are available from the corresponding author upon reasonable request.

## Abbreviations

*S. haemolyticus*: *Staphylococcus haemolyticus*
MRI: Magnetic resonance imaging
CSF: Cerebrospinal fluid:
CoNS: Coagulase-negative staphylococcus
